# The evidence for non-typeable *Haemophilus influenzae* as a causative agent of childhood pneumonia

**DOI:** 10.1186/s41479-017-0033-2

**Published:** 2017-06-25

**Authors:** Mary P E Slack

**Affiliations:** School of Medicine, Gold Coast Campus, Griffith University, Southport, Queensland 4222 Australia

**Keywords:** Non-typeable *Haemophilus influenzae*, *Community-acquired pneumonia*, *Children*

## Abstract

*Haemophilus influenzae* type b (Hib) was a major cause of bacterial pneumonia in children prior to the introduction of Hib-conjugate vaccines. The widespread use of Hib-conjugate vaccines has resulted in a significant decline in the number of cases of invasive Hib disease, including bacteraemic pneumonia, in areas where the vaccine has been implemented. In many countries, non-typeable *H. influenzae* (NTHI) is now the most common cause of invasive haemophilus infection in all ages. NTHI are a recognized cause of bacteraemic and non-bacteraemic pneumonia in children and in adults. Less than 10% of cases of pediatric pneumonia are bacteraemic, and children generally do not expectorate lower respiratory tract secretions, so determining the microbial cause of a non-bacteraemic pneumonia is challenging. In this commentary the evidence that NTHI is a cause of pneumonia in children is briefly reviewed.

## Background

Following the introduction of Hib-conjugate vaccines, the number of cases of invasive Hib infections, including bacteraemic pneumonia, has declined wherever the vaccine has been implemented. In many countries non-typeable *Haemophilus influenzae* (NTHI) is now the most common cause of invasive haemophilus infection in all ages [1]. The clinical spectrum of invasive NTHI infections is similar to that of Hib, in that it can cause meningitis, bacteraemia, pneumonia, and other localized invasive infections; however, NTHI is more likely to cause bacteraemia or pneumonia, and Hib was predominantly associated with meningitis in young children and acute epiglottitis in older children (>18 months) in high-income countries [2].

### NTHI as a cause of pneumonia in children

While NTHI are a well recognized cause of both bacteraemic and non-bacteraemic pneumonia in adults, the data on the relative importance of NTHI in childhood pneumonia is limited and, to some extent, conflicting [3]. This is due, in part, to the difficulty in obtaining samples to determine the cause of non-bacteraemic lower respiratory tract infections that have not been contaminated with upper respiratory tract commensals (including NTHI). Trans-thoracic fine-needle aspiration is the most reliable method, but it is only performed where there is dense peripheral consolidation and is not a routine procedure. Other less direct diagnostic techniques, including broncho-alveolar lavage (BAL) and sputum cultures may be used, but are still problematic. NTHI can be present in the upper airway in the absence of disease, and isolation of NTHI from an expectorated or induced sample of sputum in a child with clinical and radiographic evidence of pneumonia may be coincidental rather than causal. In young children it can be difficult to distinguish pneumonia from bronchiolitis [4]. The definition of pneumonia used by investigators also varies considerably, and is frequently not stated in publications, making it difficult to compare results from different studies. The Pneumococcal Vaccines Accelerated Development and Introduction Plan (Pneumo-ADIP) developed a case definition for childhood pneumonia, based on cough, respiratory difficulty and tachypnea [5] and its use would provide standardization across different studies.

### Direct evidence

Studies from Papua New Guinea in the 1980s [3] suggested that NTHI was a major cause of pneumonia in children in these communities. More recent studies of community-acquired pneumonia (CAP) in children, reviewed by Slack [6], have identified NTHI in a proportion (range 4–26%) of lung aspirates by culture and/or molecular typing.

A study from The Gambia reported the results of molecular testing of lung and pleural aspirates collected from 55 children (aged 2–59 months) with severe radiologically confirmed pneumonia [7]. There were 56 samples collected in total. A pathogen was isolated from 53/56 (95%) samples by one or more laboratory method, and 12/53 (23%) were identified as *H. influenzae*. Molecular characterisation of these 12 isolates showed that 3 were NTHI and it was suggestive that an additional 3 may also have been NTHI; however, there was insufficient DNA to permit full multilocus sequence typing. Only 1 was Hib.

Although the data is limited, there seems to be no doubt that NTHI is the cause of a small proportion of non-bacteraemic cases of CAP in children and may cause a greater proportion of such cases in developing countries. The incidence and case fatality rate of pneumonia in developing countries is tenfold higher than in developed countries [8]. This probably reflects underlying problems of poverty, malnutrition, overcrowding, and inadequate healthcare in the developing world. In such vulnerable hosts it is entirely possible that low-grade pathogens such as NTHI would cause a higher proportion of respiratory infections.

### Indirect evidence

Identification of bacteraemic pneumonia relies on isolation of the putative pathogen from a normally sterile site—usually a blood culture. Several studies have reported on pediatric invasive NTHI infections in the era of Hib-conjugate vaccination.

Collins et al. [9] reported on the follow up of invasive *H. influenzae* disease in England and Wales, which was diagnosed between 2000 and 2013. Over this period there were 1585 cases of invasive *H. influenzae* infection in children aged 1 month to 10 years, in whom NTHI caused 31–51 cases per year (0.53–0.92/100,000). Detailed clinical follow up of 214 cases of invasive NTHI infection occurring between 2009 and 2013 revealed that 52% (*n* = 111) occurred in <2 year olds and 52% (*n* = 110) had an identified comorbidity. Bacteraemic pneumonia was the most common presentation (*n* = 99, 46%). Bacteraemic pneumonia was also the most common (47%) presentation in a Canadian study [10]. In a retrospective surveillance study in the Netherlands [11], 47% of cases in children aged 7 weeks to 5 years presented with meningitis (28 of 60 cases) and approximately 25% presented with bacteraemic pneumonia. In a German study [12], 34% of pediatric cases of invasive NTHI infection presented with meningitis, 28% with septicaemia without focus, and 21% presented with pneumonia. This is similar to the findings from an Israeli study [13] where 25% of NTHI invasive infections presented with pneumonia. One possible reason for the disparity between these studies is differing blood culture practices in children presenting with high fever with or without localising foci such as a pneumonia.

Although the data from these studies is not strictly comparable, they do suggest that invasive NTHI can present as a pneumonic infection in children aged from 6 weeks, with the majority of cases occurring in children aged <4 years.

NTHI are a significant component of the nasopharyngeal microbiota and the organisms can spread contiguously from the upper respiratory tract to cause otitis media, sinusitis and pneumonia in otherwise healthy children. A preceding viral upper respiratory tract infection may facilitate the development of lower airways infection, including CAP [6]. Invasive NTHI infections, including meningitis, bacteraemia and bacteraemic pneumonia usually occur in children with underlying predisposing conditions, including immunosuppression and chronic respiratory diseases, although some cases do occur in previously healthy children. In the study reported by Kalies et al. [12], 62% of the cases of NTHI invasive infection had no known predisposing condition. By contrast, van Wessell [11] reported comorbidities in approximately 50% of the cases in children aged 1–4 years.

There is also evidence that NTHI are a major pathogen in cases of acute non-responding or recurrent pneumonia. In a retrospective study [14], 250 previously healthy children who developed either recurrent or non-responding pneumonia underwent flexible bronchoscopy with a semi-quantitative culture of BAL. NTHI was identified in 51% of the cases of recurrent community-acquired bronchopneumonia and 26% of non-responsive community-acquired bronchopneumonia, often in mixed culture [14].

Although invasive NTHI infection in children is uncommon and the organism is less virulent than Hib, NTHI can cause severe disease, particularly in neonates who are often premature, and in older children who have significant comorbidities [15]. Neonatal invasive NTHI infections are well described, with an incidence of 1.6–4.9/100,000 live births, accounting for approximately 5% of all neonatal bacterial invasive infections [15]. The majority of neonatal infections present as sepsis without focus, rather than pneumonia [15]. In the UK study [9], 16% of the cases required intensive care and 11% died. A European surveillance study [1] reported a case fatality ratio of 17.4% in <1 year olds, 9.2% in 1–4 year olds, and 5.2% in 5–14 year olds.

## Conclusions

It is clear that in countries where Hib has been virtually eliminated by the use of Hib-conjugate vaccine, NTHI has emerged as the most common cause of pediatric *H. influenzae* infections, including pneumonia. A vaccine effective against NTHI could be of value in preventing these infections. The 10-valent pneumococcal vaccine (PHid-CV; Synflorix, GSK Biologicals, Rixensart, Belgium) uses *H. influenzae* outer membrane lipoprotein D as its carrier protein, which is conserved among strains of *H. influenzae*. Immunisation results in high IgG antibody concentrations against protein D, but has no effect on nasopharyngeal NTHI colonisation or *H. influenzae* density [16]. Its efficacy against invasive NTHI infections has not yet been demonstrated [15], although a recent report suggests PHiD-CV10 may reduce the prevalence of suppurative otitis media in children at high risk of severe middle ear disease [17]. Whole genome sequencing of 20 nasal or nasopharyngeal NTHI isolates from a cohort of Aboriginal and Torres Strait Islander children in Australia, selected to represent common NTHI genotypes, revealed that 5 of 20 NTHI genomes lacked the *hpd* genes that encode Protein D [18]. The challenge is to develop a vaccine that overcomes the marked heterogeneity and phase variability of NTHI. Because NTHI now cause as much acute middle ear disease as pneumococci [19], and are the dominant microbe in chronic and recurrent middle ear disease worldwide, an effective NTHI vaccine could be cost-effective in many pediatric populations. Further studies to identify the specific risk factors that predispose children to invasive NTHI infection should be undertaken [15]. Accurate identification of NTHI and systematic reporting of cases of pediatric NTHI infections would be of great value in establishing the true role of NTHI in pediatric CAP. Surveillance, coupled with improved diagnostic techniques to identify the microbiological causes of non-bacteraemic pneumonia, is needed to fully understand the role of NTHI in pediatric pneumonia.

## Abbreviations

CAP: Community-acquired pneumonia
Hib: *Haemophilus influenzae* type b
NTHI: Non-typeable *Haemophilus influenzae*
